# Hydrocortisone Promotes Differentiation of Mouse Embryonic Stem Cell-Derived Definitive Endoderm toward Lung Alveolar Epithelial Cells

**DOI:** 10.22074/cellj.2021.7929

**Published:** 2021-03-01

**Authors:** Mohammad Reza Mokhber Dezfouli, Sirous Sadeghian Chaleshtori, Azadeh Moradmand, Mohsen Basiri, Hossein Baharvand, Yaser Tahamtani

**Affiliations:** 1. Department of Internal Medicine, Faculty of Veterinary Medicine, University of Tehran, Tehran, Iran; 2. Institute of Biomedical Research, Faculty of Veterinary Medicine, University of Tehran, Tehran, Iran; 3. Department of Stem Cells and Developmental Biology, Cell Science Research Center, Royan Institute for Stem Cell Biology and Technology, ACECR, Tehran, Iran; 4. Department of Developmental Biology, University of Science and Culture, ACECR, Tehran, Iran

In this article which was published in Cell J, Vol 20, No 4, winter 2019, on pages 469-479, the authors regret to acknowledge that we failed to mention in our article that a patent based on this study had been filed by Royan Institute and Tehran University with S.S.C., M.R.M.D., H.B., and Y.T. as inventors.

The authors would like to apologies for any inconvenience caused.

